# Lhermitte-Duclos disease: an extremely rare cerebellar tumor

**DOI:** 10.11604/pamj.2017.28.51.12198

**Published:** 2017-09-20

**Authors:** Salah Bellasri, Soufiane Belabbas

**Affiliations:** 1 Neuro-Imaging Department, Fifth Military Hospital, Guelmim, Morocco

**Keywords:** Lhermitte-duclos, dysplastic cerebellar gangliocytoma, cerebellar hamartoma

## Image in medicine

A 42-year-old female presented with complaints of mild occipital headaches of severe months duration. There were no cutaneous lesions or significant family history suggesting any genetic disease. On examination, she did not have any papilloedema, dymetria, dysdiadokokinasia or ataxia. Her cranial nerve examination was normal. On non-enhanced computed tomogram (CT) showed a mildly hyperdense well circumscribed lesion, in the left upper cerebellar hemisphere with no bone involvement (not schown). MRI showed an intra-axial lesion measuring 3.7cm × 3.5cm × 2.5cm in relation to the tentorium cerebelli and lateral sinus which was hypointense on T1-weighted image (T1WI) (A), with the characteristic striated appearance or “tiger-striped” appearance on T2-weighted image (T2WI) (B), there was no apparent contrast enhancement (C). This lesion was hyperintense in diffusion (D) and in fluid attenuation inversion recovery (FLAIR) (E). There was no extension into the internal auditory canal. There was no compression of the brainstem and ventricular system. Proton (¹H) MR spectroscopy study (MRS) revealed normal N-acetyl-aspartate (NAA)/Creatine (Cr) ratio (1.48), moderately decreased choline (cho)/Cr (0.76) (F). An abdominal ultrasound was performed in order to evaluate the intraabdominal organs and found to be normal. She has been under routine follow-up for 3 years. On the last control, the brain MRI showed lesion stability. Given the well-tolerated nature of her headaches and the stability of the lesion, the staff of the neurosurgeons indicated a routine control without surgery.

**Figure 1:**
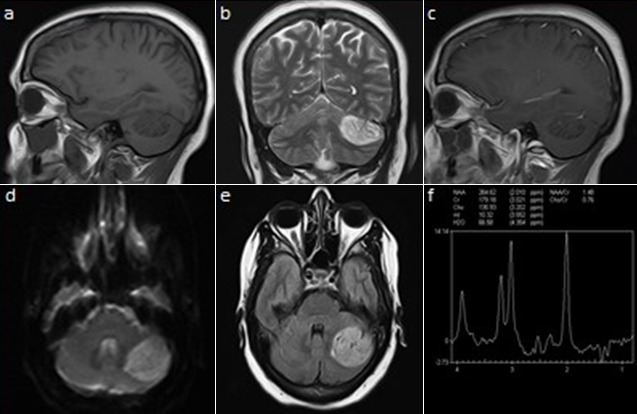
Head MRI: (A) sagittal T1-WI; (B) coronal T2-WI; (C) sagittal post contrast T1-WI; (D) diffusion WI; (E) FLAIR; (F) MRS: demonstrate left upper cerebellar hemisphere intra axial lesion, well defined, with striated appearance on T2-WI, which not enhance after gadolinium injection. This lesion appear with high intensity in FLAIR and DWI and moderately decreased choline (cho)/Cr ratio

